# Agreement Between High-Risk Human Papillomavirus Testing in Paired Self-Collected and Clinician-Collected Samples from Cervical Cancer Screening in Spain

**DOI:** 10.3390/cancers17010063

**Published:** 2024-12-29

**Authors:** Raquel Ibáñez, Esther Roura, Francisca Morey, Miguel Andújar, Miquel Ángel Pavón, Amelia Acera, Laia Bruni, Silvia de Sanjosé

**Affiliations:** 1 Cancer Epidemiology Research Programme, Catalan Institute of Oncology—Bellvitge Biomedical Research Institute (IDIBELL), L’Hospitalet de Llobregat, 08908 Barcelona, Spain; eroura@iconcologia.net (E.R.); fmorey@iconcologia.net (F.M.); mpavon@iconcologia.net (M.Á.P.); lbruni@iconcologia.net (L.B.); 2 Centro de Investigación Biomédica en Red de Epidemiología y Salud Pública (CIBERESP), 28029 Madrid, Spain; desanjose.silvia@gmail.com; 3 Pathology Department, Complejo Hospitalario Universitario Insular-Materno Infantil, 35016 Las Palmas de Gran Canaria, Spain; mandsan@gobiernodecanarias.org; 4 Atenció a la Salut Sexual i Reproductiva (ASSIR), SAP Cerdanyola-Ripollet, Institut Català de la Salut, Ripollet, 08291 Barcelona, Spain; amelia.acera@gmail.com; 5 ISGlobal, 08036 Barcelona, Spain

**Keywords:** HPV, self-sampling, agreement, concordance, Spain, cervical cancer, screening, Ct values

## Abstract

**Background**: Implementing self-sampling (SS) in cervical cancer screening requires comparable results to clinician-collected samples (CCS). Agreement measures are essential for evaluating HPV test performance. Previous studies on non-paired samples have reported higher viral cycle threshold (Ct) values in SS compared to CCS, affecting sensitivity for detecting cervical intraepithelial neoplasia grade 2 or worse (CIN2+). **Objectives:** We aimed to evaluate the agreement of high-risk (hr)HPV testing results between SS and CCS using paired samples and to explore differences in Ct values. **Methods**: Women aged 30 to 65 years attending cervical cancer screening in two regions of Spain were invited to participated in this study. For each woman there was: CCS collected during the screening visit using liquid-based cytology and cytobrush, and a SS using a brush at home one month later. A PCR-based assay was used for hrHPV detection. Agreement in hrHPV results among both samples, Ct value differences, and their association with screening outcomes were analyzed. **Results**: This study included 981 women with paired samples. SS had a higher hrHPV prevalence than CCS (overall ratio of 1.3). Positive agreement for all hrHPV genotypes, HPV16, HPV18, and other hrHPV types were 85%, 91.3%, 66.7%, and 83.3%, respectively. Negative agreement was >95% for all results. Median Ct values was slightly higher in SS than in CSS (32.9 vs. 30.6, *p* = 0.02). Seven CIN2+ cases HPV positive were detected by both methods. One CIN3 case was missed by SS. **Conclusions**: This study showed a good agreement between SS and CCS for hrHPV testing in a routine screening in Spain. Despite the slightly higher Ct values for SS, no significant impact on sensitivity could be determined due to the low incidence of CIN2+ cases. Further research on larger paired samples is needed to assess the implications of Ct values on test sensitivity.

## Simple Summary

This study is the first in Spain to directly compare HPV detection in self-collected and clinician-collected samples in paired samples from women aged 30–65 who are regularly screened. The findings reveal that self-sampling is a promising alternative to traditional clinician-collected methods for cervical cancer screening, showing good agreement in detecting high-risk human papillomavirus (HPV), particularly HPV16, which is crucial in identifying precancerous conditions. However, the study also highlights that self-collected samples may have slightly weaker viral amplification signals, which could affect the accuracy of detecting HPV in cases close to the detection threshold. This research underscores the potential of self-sampling to increase accessibility and participation in cervical cancer screening programs but also points to the need for further studies in paired collected samples to ensure its effectiveness in identifying individuals at risk of cervical precancer.

## 1. Introduction

Cervical cancer remains a significant public health concern globally, despite advances in screening and vaccination. In Spain, cervical cancer continues to have a substantial impact, with the age-standardized incidence rate in 2022 estimated at 5.4 cases per 100,000 women (crude rate 8.5) and an age-standardized mortality rate of 1.6 [1], being the eleventh leading cause of cancer in women of all ages.

Cervical screening coverage in Spain varies by age, with the highest rates observed between 25 and 65 years, the target age group for the cervical cancer screening program. According to the European Survey of Health in Spain in 2020, 72% of women aged 25 to 65 had a cervical cytology in the past three years, and 80% in the past five years [2]. It is estimated that 9,380,239 sexually active women aged 25 to 65 will undergo cervical cytology every three years in Spain, representing 3,583,145 women annually [2]. However, it is noteworthy that in 2020, when the survey was conducted, the predominant cervical cancer screening recommendation in Spain was to perform a cervical cytology every three years on an opportunistic basis. Currently, following the modification of the cervical cancer screening program of the National Health System (Order SCB/480/2019), regions are transitioning to a population-based screening program, using liquid-based cytology for primary screening in women aged 25–29/34 years and detection of carcinogenic genotypes of human papillomavirus (hrHPV) every five years starting at age 30/35, with cytological triage for screen positives [3]. Screening ends at 65 years. Most regions with HPV testing use risk-based clinical management algorithms, with limited genotyping [4].

In Spain, the systematic HPV vaccination for adolescent girls was implemented in 2008, with the vaccination age set at 12 years. In 2018, the recommendation was extended to certain conditions with a higher risk of developing anogenital cancer from HPV, such as women treated for cervical intraepithelial neoplasia grade 2 or 3 (CIN2-3). In 2022, systematic vaccination was extended to adolescent boys aged 11 to 12 years [5,6]. In July 2024, a single-dose schedule was approved up to age 25, with a two-dose schedule for those aged 26 and older, except for individuals who are immunocompromised or undergoing treatment for HSIL/CIN2-3, who will continue with the three-dose schedule [5]. Since the start of the program, the overall HPV vaccination coverage in Spain has ranged between 70–80%. In 2023, the national coverage for the full vaccination schedule at age 15 was 86%, and the coverage for one dose was 91% [7].

Although the incidence of cervical cancer has declined in many countries due to the implementation of regular screening programs and the introduction of HPV vaccination, the disease remains a leading cause of cancer-related deaths, especially among underscreened or never screened women [8,9]. Nowadays, many scientific societies and organizations worldwide, including the World Health Organization (WHO), recommend using the detection of hrHPV as primary screening test rather than cervical cytology in women aged 30 years and above [4,10,11,12,13]. This shift has facilitated the introduction of new sample collection methods, such as cervico-vaginal self-sampling (SS). Numerous studies have evaluated the acceptability of SS among women in different regions and countries worldwide, demonstrating its feasibility and high acceptance among participants [14,15]. In most of the studies, women reported ease of use, reduced embarrassment, increased privacy, enhanced comfort, and overall convenience [15,16,17]. SS can be used to improve screening coverage [18] particularly for the underscreened women in whom the majority of cervical cancers are diagnosed [9,19].

A critical aspect is that to confidently implement SS, results need to not inferior with those seen when using CCS. A systematic review including data from 56 studies and 25 randomized controlled trials (RCT) conducted in under- or never screened women showed that the detection of cervical intraepithelial neoplasia grade 2 or worse (CIN2+) through HPV testing on SS samples versus CCS was comparable, provided HPV DNA assays were based on a highly sensitive approach using a polymerase chain reaction (PCR) [20]. Further RCT and population studies conducted in regular screening populations confirmed these findings [21,22].

However, a study from the Netherlands [23], where hrHPV-based screening programs used PCR as the HPV assay with either SS or CCS, observed a 6% lower sensitivity and a 2% higher specificity for cervical intraepithelial neoplasia grade 3 or worse (CIN3+) in unpaired SS compared to clinician-based hrHPV testing. The authors suggested the detection threshold values (the Ct values) for both sampling approaches may need to be adapted to the sampling approach, as SS samples had, on average, higher Ct values [23].

The main objective of the study here presented was to evaluate the agreement of HPV test results between SS and CCS using paired samples and to explore the differences in Ct values between both samples. This study is part of a wider investigation that evaluated the implementation of SS in cervical cancer screening in Spain [17].

## 2. Materials and Methods

This is a cross-sectional study included within an RCT carried out in 1428 women aged 30–65 years, from Catalonia and Canary Islands (Spain), recruited at cervical cancer screening clinics from November 2018 to May 2021, to evaluate the acceptability of SS. More information about the trial has been described elsewhere [17]. Briefly, all participants were not pregnant, with no history of cervical disease, and were not hysterectomized. Sociodemographic information was collected through a self-completed questionnaire, including the date and country of birth, nationality, educational level, and data on previous history of cervical cancer screening, among other information, and an acceptability questionnaire was completed when they self-collected a sample at home [17]. All participating women had a liquid-based cervical sample collected by a clinician during their screening visit. In addition, the clinician explained how to use the SS device, provided a leaflet with pictorial instructions, and gave them a SS kit to take home, along with instructions to return the sample to the health center one month after the initial visit.

**Figure 1:**
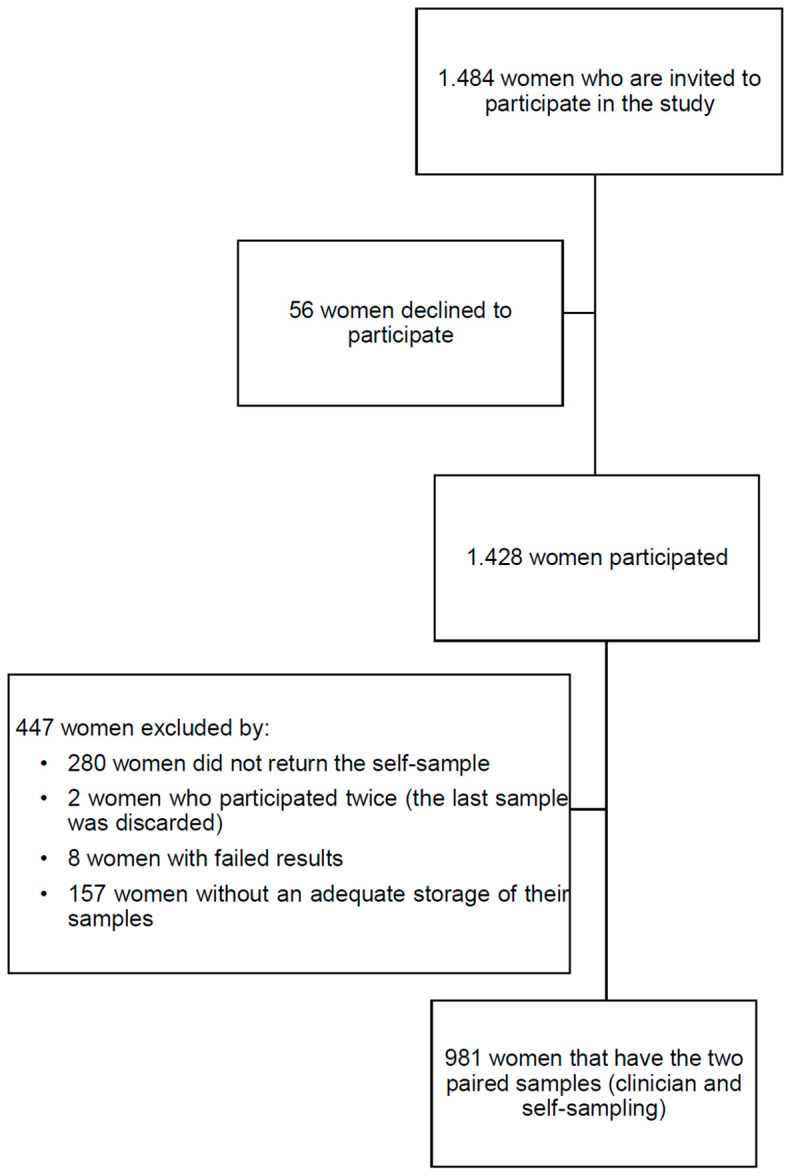
Study flowchart.

A total of 981 women with valid paired samples (CCS and SS) were included in this study (Figure 1).

### 2.1. Self-Sampling Device

The self-sample was collected using the Rovers Medical Devices Evalyn Brush (Rovers Medical Devices B.V, Oss, The Netherlands). It is a dry self-sampling device. In 2018, when the study was approved and initiated, the Evalyn Brush was the most established and evidence-supported self-sampling device. It was validated for high-risk HPV detection across multiple PCR assays and is widely used in research [24,25]. Additionally, it was included in the national cervical screening program of the Netherlands [21] and served as the comparator device in the 2018 VALHUDES protocol [26]. Its sample stability for up to 32 weeks at temperatures ranging from 4 °C to 30 °C ensured reliable results [27], making it particularly suitable for multicenter studies where sample processing may be delayed compared to a clinical diagnostic setting.

At the laboratory, the Evalyn brush tips were detached from the rest of the brush using disposable tweezers and transferred to a 15 mL tube prefilled with 4.5 mL of ThinPrep PreservCyt (Hologic Inc., Marlborough, MA, USA) for samples from Catalonia and SurePath Preservative Fluid (Becton, Dickinson, and Company (BD) Franklin Lakes, NJ, USA)) for samples from the Canary Islands. These tubes were vortexed two times for 15 s, aliquoted into 1.5 mL tubes, and frozen at −80 degrees Celsius. Only one aliquot for each woman was stored in the refrigerator until HPV detection.

### 2.2. HPV Test

Detection of hrHPV of all samples from Catalonia was carried out at the Infections and Cancer Laboratory at the Catalan Institute of Oncology in Barcelona; samples from the Canary Islands were processed at the Pathological Department of Complejo Hospitalario Universitario Insular Materno Infantil of Las Palmas de Gran Canaria.

All samples were processed using the Roche Cobas 4800 HPV test (Roche Molecular Diagnostics, Pleasanton, CA, USA). The Cobas HPV test is an in vitro qualitative detection technique for hrHPV DNA by polymerase chain reaction (PCR) amplification and can detect a total of 14 hrHPV subtypes, including HPV 16, 18, 31, 33, 35, 39, 45, 51, 52, 56, 58, 59, 66, and 68, and provides cycle threshold (Ct) value for three separate channels: HPV 16 and HPV 18, and the pooled results of the other 12 subtypes in the assay (hereafter referred to “others hrHPV non-HPV16/18”). Ct value is the PCR cycle at which the reaction signal (amplification of the viral DNA) crosses the background noise level and becomes positive. It is used as a proxy for viral load although it is dependent on the cellularity of the sample. The system’s Ct cut-off values of HPV16, HPV18, and non-16/18 hrHPV were 40.5, 40.0, and 40.0 and the cut-off for the overall hrHPV was 40.5 PCR cycles. The Cobas HPV test was carried out according to the manufacturer’s protocol. The amplification and detection stage were interpreted using the software supplied with the Cobas 4800 platform. The assay uses a human β-globin gene, as an internal control to identify false negatives caused by inadequate DNA or failed PCR.

### 2.3. Data Collection

Women were screened by cytology as the primary screening test. The hrHPV tests (CCS and SS) were performed in the context of this study. Screening cytology and histological results related to baseline hrHPV results were retrieved from the primary care clinical history of the participants. Women with negative cytology were recommended to re-screen in three years while women with low-grade intraepithelial lesion (LSIL) or worse (LSIL+) underwent colposcopy. In case of atypical squamous cells of uncertain significance (ASC-US), a reflex HPV test was recommended, and in case of hrHPV positivity, women were referred to colposcopy. Women with an ASC-US hrHPV negative were referred to regular screening.

Women with any positive cytology or any with discordant hrHPV results had their clinical history followed-up until December 2023. Histology or clinical information was used to classify the final disease status.

Viral Ct values were collected for HPV16, HPV18, and other hrHPV non-HPV16/18 in both samples (CCS and SS) using the software Cobas 4800 Archive Viewer, given that they are not values obtained automatically along with the hrHPV test result. An overall viral Ct value was calculated as the same Ct value of the respective channel in case of single channel positivity or to the smallest Ct value in case of multiple channel positivity [23]. β-globin gene values were also collected for all samples in Catalonia, but only for HPV positive samples in Canary Islands. The β-globin gene values in the HPV-negative samples from the Canary Islands could not be retrieved, as it is no longer a default value provided with the test result, and retrospective recovery was not possible for those samples.

Sociodemographic data and hrHPV test results (CCS and SS) were encrypted using Research Electronic Data Capture (REDCap) and hosted at Catalan Institute of Oncology [28,29].

### 2.4. Statistical Analyses

Age was categorized into 10-year groups for sociodemographic description and into two age groups for the rest of the analyses, <50 years (premenopausal) and >50 years (post-menopausal). Continuous variables following a normal distribution were presented as mean values with a 95% confidence interval (CI), while non-normally distributed variables were displayed using median values with interquartile range (IQR). Categorical variables were presented as percentages.

Cytological results were categorized as negative, ASC-US/LSIL and atypical squamous cells that cannot exclude high-grade (ASC-H) or worse (ASC-H+) including cytological results of ASC-H, high-grade squamous intraepithelial lesion (HSIL), and atypical glandular cells (AGC).

Percent of positive and negative agreement, positive and negative concordance, test positivity rate ratio, observed agreement, and concordance performed by Cohen’s Kappa Index [30], with 95% CI between CCS and SS, were calculated for the overall results (positive/negative) and for HPV16, HPV18, and other hrHPV non-HPV16/18 genotypes [31]. Positive agreement referred to the proportion of samples that were positive in both methods relative to those positive in at least one, while positive concordance refers to the proportion of samples positive in both methods relative to all samples positive in either method. Negative agreement and concordance were defined similarly [31]. Positive ratio was calculated as the proportion of positive results in SS relative to the proportion of positive results in CCS [31].

Among hrHPV positive women, we compared viral Ct values of Cobas 4800 HPV Test in SS and CCS using the Kruskal–Wallis test, since variables did not follow normal distribution, and the Wilcoxon Signed-Rank test for paired data. A histogram was created with viral Ct values of positive samples for SS and CCS. Differences between CCS and SS Ct values were also calculated by age category and cytological results. We applied linear regression to check whether the difference in viral Ct values between SS and CCS was mediated by age and cytology results.

All samples with an invalid hrHPV result were excluded from the analyses. All statistical tests were two-tailed, and *p*-values below 0.05 were considered statistically significant.

Data analyses were carried out with R software version 4.1.0 (R Core Team (2015). R: A language and environment for statistical computing. R Foundation for Statistical Computing, Vienna, Austria (https://www.R-project.org/ accessed on 15 January 2021).

### 2.5. Ethics Approval

The main project was approved by the ethical committees of Bellvitge University Hospital (PR223/17), University Institute for Primary Health Care Research (IDIAP) Jordi Gol i Gurina (P18/099), and Complejo Hospitalario Universitario Insular Materno Infantil of Las Palmas de Gran Canaria (2018-178-1). All the women who accepted to participate in this study signed informed consent.

## 3. Results

A total of 981 women, with paired SS and CCS, were included in this study with a mean age of 45 years. Most women were Spanish (86.1%) and 60.3% were recruited in Catalonia. Almost all participants had undergone previous cervical cancer screening tests (98.9%), with 66.2% having been screened within the last 3–4 years, and 64.5% reporting having undergone at least five screening tests or more during their lifetime (Table 1).

Agreement and concordance statistics for hrHPV test results between SS and CCS are shown in Table 2. Among the 981 women, five samples had invalid results (four in self-samples and one in the professional samples). SS were more likely to be hrHPV positive than CCS (ratio of 1.3, 95% CI: 1.1–1.5). The overall percentage of positive and negative agreement was 85% (95% CI: 78.2–91.8%) and 95.1% (95% CI: 93.7–96.5%), respectively. The overall agreement between SS and CCS was 93.9% (95% CI: 92.4–95.4%), with a Kappa value of 0.72 (95% CI: 0.7–0.8).

The agreement in positivity by the HPV16, HPV18, and other hrHPV channels was 91.3%, 66.7%, and 83.3% respectively (Table 2), while the positive concordance was 65.6%, 25%, and 59.7% respectively. Note that there were very few HPV18 positive samples. The agreement in negativity and the channel negative concordance were high for all hrHPV results.

The prevalence of hrHPV in CCS in the Canary Islands was higher than in Catalonia (13.9% vs. 9% respectively, *p* = 0.02, Supplementary Table S1), although no differences were found in SS (Canary Islands HPV prevalence 15.9% vs. 12.3% in Catalonia, *p* = 0.12, Supplementary Table S1). No differences in overall agreement between samples collected in SurePath (Canary Islands) and ThinPrep (Catalonia) were found (92.8% vs. 94.7% respectively; *p* = 0.27, Supplementary Table S1).

The hrHPV results analyzed hierarchically for both SS and CCS, stratified by cytology results are shown in Table 3. In this hierarchy, HPV16 infections were first considered, then HPV18 infections and finally others hrHPV non-HPV16/18. Most importantly no HPV16 positive ASC-H+ cases were missed by any test. One ASC-H+ case was missed by SS. It was a case with ASC-H in the cytology and positive for hrHPV non-HPV16/18 on CCS. One case of AGC was missed by both sampling methods, lately shown to be of endometrial origin.

Table 4 shows the viral HPV Ct median values in SS and CCS by age groups and cytology results. Globally, median viral Ct values was slightly higher for SS than for CCS (32.9 vs. 30.6 respectively, *p* = 0.02). Among the samples of women under 50 years SS showed higher median Ct values compared to CCS (32.8 vs. 30.0 respectively; *p* = 0.02). However, for women above 50 years, no significant difference was observed between SS and CCS (34.8 vs. 33.6 respectively; *p* = 0.57). The histogram showing the frequency of viral Ct values for CCS and SS samples (Supplementary Figure S1) also indicated a trend for the Ct values of SS to be higher than those of CCS. Viral median Ct values was not different by cytology results in any of the sampling methods.

In the linear regression analysis, no significant effects on HPV detection between collection methods, age and cytology results were found (Table 4).

Among the 976 paired samples, 1.6% (N = 16) were hrHPV negative in SS but positive in CCS. Among these discordant samples, 37.5% (6/16) had a Ct value ≥38 (near the assay cut-off of 40) and 43.8% (7/16) had Ct values ranging between 33.1 and 36.8. The remaining three samples (18.8%) had Ct values between 26.5 and 27.3.

## 4. Discussion

The agreement and concordance between SS and CCS in paired samples were evaluated in a cohort of women aged 30–65 years attending a routine cervical cancer screening in Spain. Samples were collected as part of the regular screening process, with SS performed at home by the women without clinician assistance. Results showed a high level of agreement and concordance between the two sample collection methods, both overall and across different HPV genotypes. The only exception was observed in the few HPV18 samples, where the positive agreement was lower compared to other genotypes. These findings support the use of SS as a reliable alternative to CCS in cervical cancer screening, particularly in routine screening.

Our study contributes to the growing body of literature supporting the use of SS for hrHPV testing. Our findings are consistent with previous research, including a study from Kaiser Permanente Northern California (N = 144 paired collections) [32], which reported an overall positive agreement of 84.2% between SS and CCS with channel agreement rates of 90.3% for all HPV genotypes. The study also found high agreement for various HPV genotypes, with 97.8% agreement for HPV16, 99.3% for HPV18 and 92.4% for other hrHPV types. A similar study in Australia (303 paired samples) [33] also found comparable agreement rates between SS and CCS. In line with the meta-analysis previously mentioned in the introduction, which includes 26 studies with over 10,000 participants [31], a positive concordance of 59.3% (95% CI: 44.4–73.5%) and a negative concordance of 85.6% (95% CI: 77.9–91.8%) were reported. These findings, along with our study, highlight the use of SS as a tool for HPV testing in screening populations.

According to the authors of this meta-analysis, evaluating agreement/concordance parameters could also be used to validate new self-sample collection devices intended for use with a previously validated HPV test for self-sampling. This approach would eliminate the need for larger validation studies requiring histological confirmation, thus reducing both time and costs, and accelerating the validation process [31].

While agreement/concordance measures are essential for evaluating HPV test performance, assessing Ct values can provide valuable additional insights. Some studies have found that there is a linear correlation between the Ct values and the logarithm of HPV viral copies in the sample. Thus, a higher initial number of viral copies is associated with lower Ct values, and a higher viral load is linked to a greater presence of lesions [34,35,36]. Nevertheless, our study confirms the findings of Inturrisi et al. [23] in the Netherlands, showing that Ct values for SS were significantly higher than for CCS. The Dutch study reported a mean Ct difference of 2.73 (95% CI: 1.75–3.72) for CIN2 and 3.59 (95% CI: 3.03–4.15) for CIN3+, with a relative sensitivity of 0.9 (95% CI: 0.90–0.97) [23]. In our study, one CIN3 case missed by SS had a Ct value of 35.2 in the CCS sample.

Eight women were diagnosed with CIN2+ (two cases of CIN2, five cases of CIN3, and one case of infiltrating squamous cell carcinoma). All but one CIN3 case tested HPV-positive using both sampling methods, with HPV16 detected in the carcinoma case and one CIN2 case, while all eight CIN2+ cases were also positive for other hrHPV types excluding HPV16/18. To draw more accurate conclusions regarding the relationship between higher Ct values in SS and potential sensitivity loss, larger studies involving paired samples in a screening context would be necessary.

Although the median viral Ct values in SS were slightly higher than those found in CCS, the study by Inturrisi et al. found no differences among age groups [23]. We observed variations in median Ct values among women under 50 years of age; however, linear regression analysis did not reveal statistically significant age-related differences underscoring the contribution of age associated with sampling methods.

In the Netherlands, 46.3% of the participants cited doubts about obtaining the SS correctly [16], while in our study 29.3% of the participants showed doubts about whether they had correctly taken the sample [17]. The proportion of SS with an invalid result was very low (4/981, 0.4%) in our study and lower than the 1.1% (95% CI 0.4–2.1%) observed in a metanalysis conducted in 20 RCTs [37]. This low rate of invalid results should reassure women and providers about the quality of SS, although adequate health education remains crucial for obtaining reliable results [17].

A strength of our study was that it was conducted among regular screening users providing paired samples to comply with an agreement analysis. HPV SS was used in the context of primary screening, and women performed it at home without clinician assistance. Furthermore, we included a detailed analysis of Ct values, a measure that is not always assessed but can provide valuable insights into viral load and test sensitivity. All Ct values from HPV-positive samples, both from SS and CCS, were included. Only one CCS sample did not have a Ct value available, as Ct values are not automatically provided with the HPV result and must be extracted separately. Additionally, the use of two liquid-based cytology media available on the market (SurePath and ThinPrep) and the lack of differences between them contribute to the consistency of the study’s results.

However, a limitation of our study was limited number of cases of CIN2+ due to its focus on women undergoing routine screening, correlated by the low incidence rate in our country [1]. This limitation hindered our ability to conduct a comparative analysis of sensitivity and specificity between SS and CCS. Nonetheless, although our study was not primarily designed to assess the sensitivity and specificity of SS compared to CCS, but rather to evaluate the agreement of results, our data are consistent with the realities observed in clinical practice within the screening context in Spain [38], which strengthens the applicability of our results to real-world settings.

Despite the limitations, this study provides evidence supporting the use of SS for hrHPV testing in routine cervical cancer screening. The high agreement between SS and CCS, and the low rate of invalid results, suggest that SS can be a reliable alternative to CCS. This could increase coverage and accessibility of screening, especially in populations with barriers to attending a clinic.

## 5. Conclusions

In conclusion, our study confirmed that hrHPV testing on SS demonstrated a good concordance, in terms of agreement parameters, with CCS in population-based screening of women aged 30 to 65 years. However, SS tends to have a weaker amplification signal (higher Ct values in the PCR assays) compared to CCS. This highlights the need for further investigation into the implications of Ct values in SS through larger paired samples studies to determine whether these differences may indicate a potential loss of sensitivity.

## Figures and Tables

**Table 1:** Demographic characteristics of study participants.

	N = 981 Women	
	N	(%)
**Age (in years)**		
<30	18	(1.8)
30–39	313	(31.9)
40–49	313	(31.9)
50–59	270	(27.5)
≥60	67	(6.8)
**Area**		
Canarias	389	(39.7)
Catalunya	592	(60.3)
**Country of birth**		
Spain	845	(86.1)
Outside Spain	136	(13.9)
**Any previous screening test**		
No	11	(1.1)
Yes	967	(98.9)
Missing	3	
**Last self-reported screening test among ever tested**		
<3 years	301	(31.7)
3–4 years	629	(66.2)
>5 years	20	(2.1)
Missing	31	-
**Number of self-reported screening tests in lifetime among ever tested**		
1	30	(3.2)
2 to 4	304	(32.4)
5 to 7	241	(25.7)
8 to 10	169	(18.0)
>10	195	(20.8)
Missing	42	-

**Table 2:** Agreement and concordance statistics between self-sampling and clinician samples by hrHPV results.

		Overall hrHPV Results	HPV16	HPV18	Other hrHPV Non-HPV16/18
	P1/P2	91	21	2	80
	N1/P2	43	9	5	38
	P1/N2	16	2	1	16
	N1/N2	826	944	968	842
Positivity ratio	N	1.3	1.3	2.3	1.2
	95% CI	1.1–1.5	0.9–1.9	0.9–4.8	1.0–1.5
Positive agreement	%	85.0	91.3	66.7	83.3
	95% CI	78.2–91.8	79.8–1.0	13.4–100.0	75.8–90.8
Negative agreement	%	95.1	99.1	99.5	95.7
	95% CI	93.7–96.5	98.5–99.7	99.1–99.9	94.4–97.0
Positive concordance	%	60.7	65.6	25.0	59.7
	95% CI	52.9–68.5	49.1–82.1	0.0–55.0	51.4- 68.0
Negative concordance	%	93.3	98.8	99.4	94.0
	95% CI	91.6–94.9	98.1–99.5	98.9–99.9	92.4–95.6
Agreement	%	93.9	98.8	99.4	94.5
	95% CI	92.5–95.4	98.2–99.5	98.9–99.9	93.0–95.9
Kappa coefficient	N	0.72	0.8	0.4	0.7
	95% CI	0.7–0.8	0.7–0.9	0.3- 0.5	0.7–0.8
hrHPV prevalence in clinician samples	%	11	2.4	0.3	9.8
	95% CI	9.1–13.1	1.5–3.5	0.1–0.9	8.0–11.9
hrHPV prevalence in self-sampling	%	13.6	3.1	0.7	12.1
	95% CI	11.5–15.9	2.1–4.4	0.3–1.5	10.1–14.3

CI: confidence interval; HPV: human papillomavirus; hrHPV: high-risk HPV types; P: hrHPV positive; N: hrHPV negative; 1: Clinician-collected samples; 2: Self-sampling.

**Table 3:** Hierarchical self-sampling results compared to clinician-collected samples result by cytology results.

		Clinician-Collected Samples Result (Hierarchical) *				
Cytology Result		HPV16	HPV18	Others hrHPV Non HPV16/18	HPV Negative	Total
**Negative cytology**						
	**Self-sampling result (hierarchical)**					
	HPV16	11	0	1	6	18
	HPV18	0	0	0	4	4
	Other hrHPV non-HPV16/18	0	0	37	27	64
	HPV Negative	2	1	9	764	776
	Total	13	1	47	801	862
**ASC-US/LSILcytology**						
	**Self-sampling result (hierarchical)**					
	HPV16	9	0	1	1	11
	HPV18	0	0	1	0	1
	Other hrHPV non-HPV16/18	0	0	22	5	27
	HPV Negative	0	0	3	35	38
	Total	9	0	27	41	77
**ASC-H+ cytology**						
	**Self-sampling result (hierarchical)**					
	HPV16	1	0	0	0	1
	HPV18	0	1	0	0	1
	Other hrHPV non-HPV16/18	0	0	4	0	4
	HPV Negative	0	0	1	1	2
	Total	1	1	5	1	8
**Unsatisfactory cytology**						
	**Self-sampling result (hierarchical)**					
	HPV16	0	0	0	0	0
	HPV18	0	0	0	0	0
	Other hrHPV non-HPV16/18	0	0	1	0	1
	HPV Negative	0	0	0	9	9
	Total	0	0	1	9	10

* HPV reference standard was based on hrHPV results of clinician—collected samples analyzed hierarchically by cancer risk. In this hierarchy, HPV16 infections were first taken into account, then HPV18 infections, and finally other hrHPV non-HPV16/18. Samples that agree between both methods are marked in grey color.

**Table 4:** Median of viral HPV Ct values of hrHPV-positive samples in self-sampling and clinician-collected samples by age groups and cytology results.

	Self-Sampling Result			Clinician-Collected Samples Result			*p*-Value *	Univariate Lineal Regression ^#^		
	HPV Negative	HPV Positive		HPV Negative	HPV Positive			Estimates	95% CI	*p*-Value
	N	N	Median CTs (IQR)	N	N	Median CTs (IQR)				
**Total**	843	133	32.9 (28.1–37.7)	869	106 ^†^	30.6 (27.3–35.2)	**0.02**			
**Age group (years)**										
<50	544	96	32.8 (28.4–37.7)	558	81 ^†^	30.0 (27.3–35.1)	**0.02**	Ref.		
≥50	299	37	34.8 (27.3–37.4)	311	25	33.6 (27.7–36.4)	0.57	−1.63	−3.4; 0.2	0.079
**Cytology result**										
Negative	776	86	34.8 (28.8–38.1)	801	61	33.1 (28.8–36.1)	0.14	Ref.		
Abnormal result	41	44	30.5 (25.5–35.9)	42	42	27.9 (25.5–30.3)	0.07			
ASC-US/LSIL	38	39	30.5 (25.6–36.1)	41	36	27.8 (25.5–30.5)	0.09	1.89	−0.5; 4.3	0.119
ASC-H+	3	5	30.6 (25.3–31.6)	1	6 ^†^	28.4 (27.8–29.0)	0.58	3.75	−1.2; 8.7	0.139
Unsatisfactory sample	9	1	26.1	9	1	25.8	---	0.63	−10.1; 11.4	0.908
Missing	17	2	---	17	2	---	---	−0.62	−8.3; 7.0	0.872

* Kruskal–Wallis test for median values. † There was a missing result in CT value. # Reference categories: “<50” years for age group (years) variable and “Negative” for cytology result variable. CI: confidence interval; IQR: Interquartile range; ASC-H+ included cytological results of ASC-H (5 cases), H-SIL (2 cases) and AGC (1 case); AGC: atypical glandular cells; ASC-H: atypical squamous cells cannot exclude high-grade; ASC-US: atypical squamous cells of undetermined significance; HSIL: high-grade squamous intraepithelial lesion; LSIL: low-grade squamous intraepithelial lesion; Ct: cycle threshold; HPV: human papillomavirus; hrHPV: high-risk HPV types.

## Data Availability

The data presented in this study are available on request from the corresponding author.

## Supplementary Materials

The following supporting information can be downloaded at: https://www.mdpi.com/article/10.3390/cancers17010063/s1, Table S1: Agreement and concordance statistics between self-sampling and clinician samples by regions of the study, Figure S1: Histogram of viral Cts values of hrHPV-positive samples of clinician-collected samples and self-sampling.

## References

[1] Ferlay J., Ervik M., Lam F., Laversanne M., Colombet M., Mery L., Piñeros M., Znaor A., Soerjomataram I., Bray F. (2024). Global Cancer Observatory: Cancer Today (Version 1.1). https://gco.iarc.who.int/today.

[2] INE (2020). Instituto Nacional de Estadística. España. European Survey of Health in Spain 2020. https://www.ine.es/.

[3] Ministerio de Sanidad Consumo y Bienestar Social (2019). Orden SCB/480/2019, de 26 de Abril, por la que Se Modifican los Anexos I, III y VI del Real Decreto 1030/2006, de 15 de Septiembre, que Establece la Cartera de Servicios Comunes del Sistema Nacional de Salud y el Procedimiento para su Actualización. España: Boletín Oficial del Estado, número 1, Sec 1, página 43018. https://www.boe.es/eli/es/o/2019/04/26/scb480.

[4] Torné A., Andía D., Bruni L., Centeno C., Coronado P., Cruz Quílez J., de la Fuente J., de Sanjosé S., Granados R., Ibáñez R., del Pino M., Tornè A. (2022). AEPCC-Guía: Prevención Secundaria del Cancer de Cuello del Útero, 2022. Conducta Clínica Ante Resultados Anormales de las Pruebas de Cribado.

[5] Grupo de trabajo de Recomendaciones de Vacunación frente a VPH de la Ponencia de Programa y Registro de Vacunaciones Recomendación de Vacunación Frente a VPH. Revisión de La Estrategia de Una Dosis. Comisión de Salud Pública Del Consejo Interterritorial. https://www.sanidad.gob.es/areas/promocionPrevencion/vacunaciones/comoTrabajamos/docs/VPH_recomendaciones_vacunacion_estrategia1dosis.pdf.

[6] Grupo de trabajo de Vacunación frente a VPH en varones de la Ponencia de Programa y Registro de Vacunaciones (2022). Comisión de Salud Pública Del Consejo Interterritorial Del Sistema Nacional de Salud. Ministerio de Sanidad, Octubre 2022. https://www.sanidad.gob.es/areas/promocionPrevencion/vacunaciones/comoTrabajamos/docs/Recomendaciones_vacunacion_VPHVarones.pdf.

[7] Ministerio de Sanidad Portal Estadístico. Área de Inteligencia de Gestión. SIVAMIN Informe de Evolución de Coberturas de Vacunación por Vacuna.

[8] Ibáñez R., Alejo M., Combalia N., Tarroch X., Autonell J., Codina L., Culubret M., Bosch F.X., de Sanjosé S. (2015). Underscreened Women Remain Overrepresented in the Pool of Cervical Cancer Cases in Spain: A Need to Rethink the Screening Interventions. BioMed Res. Int..

[9] Castillo M., Astudillo A., Clavero O., Velasco J., Ibáñez R., Desanjosé S. (2016). Poor cervical cancer screening attendance and false negatives. A call for organized screening. PLoS ONE.

[10] von Karsa L., Arbyn M., De Vuyst H., Dillner J., Dillner L., Franceschi S., Patnick J., Ronco G., Segnan N., Suonio E. (2015). European guidelines for quality assurance in cervical cancer screening. Summary of the supplements on HPV screening and vaccination. Papillomavirus Res..

[11] World Health Organization (2021). WHO Guideline on Self-Care Interventions for Health and Well-Being.

[12] (2021). WHO Guideline for Screening and Treatment of Cervical Pre-Cancer Lesions for Cervical Cancer Prevention. https://www.who.int/publications/i/item/9789240030824.

[13] IARC (2022). Cervical Cancer Screening. Handbooks of Cancer Prevention. https://publications.iarc.fr/604.

[14] Chao Y., McCormack S. (2019). HPV Self-Sampling for Primary Cervical Cancer Screening: A Review of Diagnostic Test Accuracy and Clinical Evidence—An Update.

[15] Nelson E.J., Maynard B.R., Loux T., Fatla J., Gordon R., Arnold L.D. (2017). The acceptability of self-sampled screening for HPV DNA: A systematic review and meta-analysis. Sex. Transm. Infect..

[16] Polman N.J., de Haan Y., Veldhuijzen N.J., Heideman D.A.M., de Vet H.C.W., Meijer C.J.L.M., Massuger L.F., van Kemenade F.J., Berkhof J. (2019). Experience with HPV self-sampling and clinician-based sampling in women attending routine cervical screening in the Netherlands. Prev. Med..

[17] Ibáñez R., Roura E., Acera A., Andújar M., Pavón M., Bruni L., de Sanjosé S. (2023). HPV self-sampling among cervical cancer screening users in Spain: A randomized clinical trial of on-site training to increase the acceptability. Prev. Med..

[18] Nishimura H., Yeh P.T., Oguntade H., Kennedy C.E., Narasimhan M. (2021). HPV self-sampling for cervical cancer screening: A systematic review of values and preferences. BMJ Glob. Health.

[19] Ibáñez R., Autonell J., Sardà M., Crespo N., Pique P., Pascual A., Martí C., Fibla M., Gutiérrez C., Lloveras B. (2014). Protecting the underscreened women in developed countries: The value of HPV test. BMC Cancer.

[20] Arbyn M., Smith S.B., Temin S., Sultana F., Castle P. (2018). Detecting cervical precancer and reaching underscreened women by using HPV testing on self samples: Updated meta-analyses. BMJ.

[21] Polman N.J., Melchers W.J.G., Bekkers R.L.M., Molijn A.C., Meijer C.J.L.M., Quint W.G.V., Snijders P.J.F., Massuger L.F.A.G., van Kemenade F.J., Berkhof J. (2019). Performance of human papillomavirus testing on self-collected versus clinician-collected samples for the detection of cervical intraepithelial neoplasia of grade 2 or worse: A randomised, paired screen-positive, non-inferiority trial. Lancet Oncol..

[22] Stanczuk G., Baxter G., Currie H., Lawrence J., Cuschieri K., Wilson A., Arbyn M. (2016). Clinical validation of hrHPV testing on vaginal and urine self-samples in primary cervical screening (cross-sectional results from the Papillomavirus Dumfries and Galloway—PaVDaG study). BMJ Open.

[23] Inturrisi F., Aitken C.A., Melchers W.J.G., Brule A.J.C.V.D., Molijn A., Hinrichs J.W.J., Niesters H.G., Siebers A.G., Schuurman R., Heideman D.A. (2021). Clinical performance of high-risk HPV testing on self-samples versus clinician samples in routine primary HPV screening in the Netherlands: An observational study. Lancet Reg. Heal. Eur..

[24] Van Baars R., Bosgraaf R.P., Ter Harmsel B.W.A., Melchers W.J.G., Quint W.G.V., Bekkers R.L.M. (2012). Dry storage and transport of a cervicovaginal self-sample by use of the Evalyn Brush, providing reliable human papillomavirus detection combined with comfort for women. J. Clin. Microbiol..

[25] Hawkes D., Keung M.H.T., Huang Y., McDermott T.L., Romano J., Saville M., Brotherton J.M.L. (2020). Self-collection for cervical screening programs: From research to reality. Cancers.

[26] Arbyn M., Peeters E., Benoy I., Vanden B.D., Bogers J., Sutter P.D. (2018). VALHUDES: A protocol for validation of human papillomavirus assays and collection devices for HPV testing on self-samples and urine samples. J. Clin. Virol..

[27] Ejegod D.M., Pedersen H., Alzua G.P., Pedersen C., Bonde J. (2018). Time and temperature dependent analytical stability of dry-collected Evalyn HPV self-sampling brush for cervical cancer screening. Papillomavirus Res..

[28] Harris P., Taylor R., Thielke R., Payne J., Gonzalez N., Conde J. (2009). Research electronic data capture (REDCap)—A metadata-driven methodology and workflow process for providing translational research informatics support. J. Biomed. Inform..

[29] Harris P., Taylor R., Minor B., Elliott V., Fernandez M., O’Neal L., McLeod L., Delacqua G., Delacqua F., Kirby J. (2019). The REDCap consortium: Building an international community of software platform partners. J. Biomed. Inform..

[30] Brennan P., Silman A. (1992). Statistical methods for assessing observer variability in clinical measures. Br. Med. J..

[31] Arbyn M., Castle P.E., Schiffman M., Wentzensen N., Heckman-Stoddard B., Sahasrabuddhe V.V. (2022). Meta-analysis of agreement/concordance statistics in studies comparing self- vs clinician-collected samples for HPV testing in cervical cancer screening. Int. J. Cancer.

[32] Qi M., Naranjo A.R., Duque A.J., Lorey T.S., Jeffrey M., Suh-burgmann B.J., Rummel M., Salipante S.J., Wentzensen N., Greene D.N. (2024). Evaluation of Pre-Analytical variables for HPV Primary Screening from Self-Collected Vaginal Swabs. J. Mol. Diagn..

[33] Saville M., Hawkes D., Keung M.H.T., Ip E.L.O., Silvers J., Sultana F., Malloy M.J., Velentzis L.S., Canfel K., Wrede C.D. (2020). Analytical performance of HPV assays on vaginal self-collected vs practitioner-collected cervical samples: The SCoPE study. J. Clin. Virol..

[34] Lyufang D., Du H., Wang C., Huang X., Qu X., Shi B., Lui Y., Zhang W., Duan X., Wei L. (2020). The effectiveness of HPV viral load, reflected by Cobas 4800 HPV-Ct values for the triage of HPV-positive women in primary cervical cancer screening: Direct endocervical samples. PLoS ONE.

[35] Song F., Du H., Wang C., Huang X., Qu X., Wei L., Belinson J.L., Wu R., CHIMUST Team (2021). The effectiveness of human papillomavirus load, reflected by cycle threshold values, for the triage of HPV-positive self-samples in cervical cancer screening. J. Med. Screen..

[36] Zhang Y., Du H., Xiao A., Zhang W., Wang C., Huang X., Qu X., Wang J., Wu R. (2022). Verification of the association of the cycle threshold (Ct) values from HPV testing on Cobas4800 with the histologic grades of cervical lesions using data from two population-based cervical cancer screening trials. Infect. Agents Cancer.

[37] Costa S., Verberckmoes B., Castle P.E., Arbyn M. (2023). Offering HPV self-sampling kits: An updated meta-analysis of the effectiveness of strategies to increase participation in cervical cancer screening. Br. J. Cancer.

[38] Bruni L., Albero G., Serrano B., Mena M., Collado J.J., Gómez D., Muñoz J., Bosch F.X., de Sanjosé S. (2023). Human Papillomavirus and Related Diseases in Spain. Summary Report. https://hpvcentre.net/index.php.

