# Supplementary material for: Agreement Between High-Risk Human Papillomavirus Testing in Paired Self-Collected and Clinician-Collected Samples from Cervical Cancer Screening in Spain
Source: Cancers (Basel). 2024 Dec 29;17(1):63. doi: 10.3390/cancers17010063 (PMC11718957; doi:10.3390/cancers17010063)

Supplementary Figure S1. Histogram of viral Cts values of hrHPV-positive samples of clinician-collected samples and self-sampling

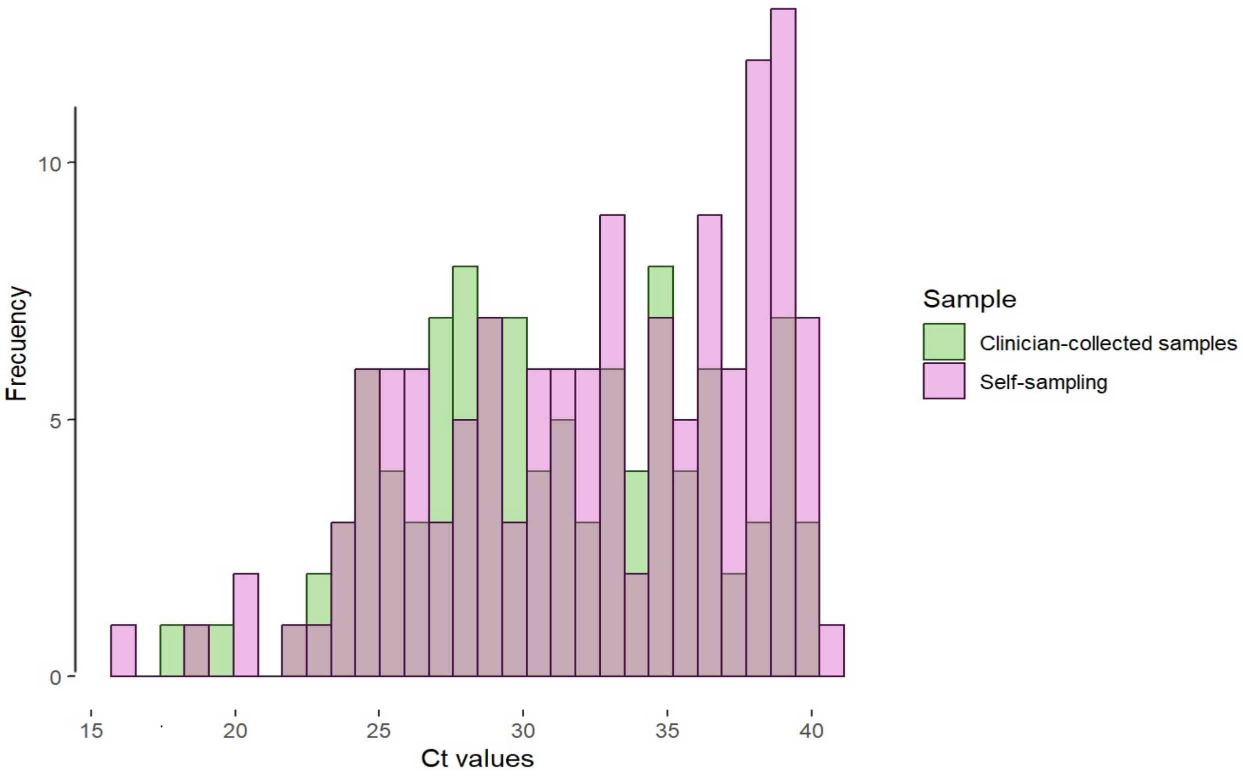

Supplement: Supplementary file 1 [file cancers-17-00063-s001.zip › Supplementary Figure S1.pdf]
