# Supplementary material for: Agreement Between High-Risk Human Papillomavirus Testing in Paired Self-Collected and Clinician-Collected Samples from Cervical Cancer Screening in Spain
Source: Cancers (Basel). 2024 Dec 29;17(1):63. doi: 10.3390/cancers17010063 (PMC11718957; doi:10.3390/cancers17010063)
Supplement: Supplementary file 1 [file cancers-17-00063-s001.zip › Supplementary Table S1.pdf]

Supplementary Table S1. Agreement and concordance statistics between self-sampling and clinician samples by regions of the study

		Catalonia (Thinprep eluted samples)	Canary Islands (SurePath eluted samples)
**Self-sampling**	**HPV positive N (%)**	72 (12.3)	62 (15.9)
	**HPV negative N (%)**	515 (87.7)	327 (84.1)
**Clinician-collected samples**	**HPV positive N (%)**	53 (9.0)	54 (13.9)
	**HPV negative N (%)**	534 (91)	335 (86.1)
**Positivity ratio**	**N**	1.4	1.1
	**95%CI**	1.1 - 1.7	0.9 - 1.5
**Positive agreement**	**%**	88.7	81.5
	**95%CI**	80.2 - 97.2	71.1 - 91.9
**Negative agreement**	**%**	95.3	94.6
	**95%CI**	93.5 - 97.1	92.2 - 97.0
**Positive concordance**	**%**	60.3	61.1
	**95%CI**	49.4 - 71.1	49.8 - 72.4
**Negative concordance**	**%**	94.3	91.9
	**95%CI**	92.3 - 96.3	89.0 - 94.8
**Overall agreement**	**%**	94.7	92.8
	**95%CI**	92.9-96.5	90-95
**Kappa coefficient**	**N**	0.72	0.72
	**95%CI**	0.6-0.8	0.6-0.8

The p-value (chi-squared) for HPV-positive self-samples between Catalonia and the Canary Islands is 0.124.

The p-value (chi-squared) for HPV-positive clinician-collected samples between Catalonia and the Canary Islands is 0.023.

The p-value (chi-squared) for differences in overall agreement between samples collected in SurePath (Canary Islands) and ThinPrep (Catalonia) is 0.27.

CI: confidence interval.
